# Epidemiologic association and shared genetic architecture between cataract and hearing difficulties among middle-aged and older adults

**DOI:** 10.1186/s40246-024-00601-z

**Published:** 2024-04-17

**Authors:** Xiayin Zhang, Shan Wang, Shunming Liu, Zijing Du, Guanrong Wu, Yingying Liang, Yu Huang, Xianwen Shang, Yijun Hu, Zhuoting Zhu, Wei Sun, Xueli Zhang, Honghua Yu

**Affiliations:** 1 Department of Ophthalmology, Guangdong Eye Institute, Guangdong Provincial People’s Hospital, Guangdong Academy of Medical Sciences, Southern Medical University, Guangzhou, China; 2 Guangdong Cardiovascular Institute, Guangdong Provincial People’s Hospital, Guangdong Academy of Medical Sciences, Guangzhou, China; 3 Centre for Eye Research Australia, Royal Victorian Eye and Ear Hospital, VIC East Melbourne, Australia; 4 Department of Ophthalmology, Guangzhou First people’s Hospital, Guangzhou, China; 5 Guangdong Provincial Key Laboratory of Artificial Intelligence in Medical Image Analysis and Application, Guangzhou, China

**Keywords:** Cataract, Hearing difficulties, Shared genetic architecture, Sensory traits, Oxidative stress

## Abstract

Age-related cataract and hearing difficulties are major sensory disorders that often co-exist in the global-wide elderly and have a tangible influence on the quality of life. However, the epidemiologic association between cataract and hearing difficulties remains unexplored, while little is known about whether the two share their genetic etiology. We first investigated the clinical association between cataract and hearing difficulties using the UK Biobank covering 502,543 individuals. Both unmatched analysis (adjusted for confounders) and a matched analysis (one control matched for each patient with cataract according to confounding factors) were undertaken and confirmed that cataract was associated with hearing difficulties (OR, 2.12; 95% CI, 1.98–2.27; OR, 2.03; 95% CI, 1.86–2.23, respectively). Furthermore, we explored and quantified the shared genetic architecture of these two complex sensory disorders at the common variant level using the bivariate causal mixture model (MiXeR) and conditional/conjunctional false discovery rate method based on the largest available genome-wide association studies of cataract (*N* = 585,243) and hearing difficulties (*N* = 323,978). Despite detecting only a negligible genetic correlation, we observe polygenic overlap between cataract and hearing difficulties and identify 6 shared loci with mixed directions of effects. Follow-up analysis of the shared loci implicates candidate genes QKI, STK17A, TYR, NSF, and TCF4 likely contribute to the pathophysiology of cataracts and hearing difficulties. In conclusion, this study demonstrates the presence of epidemiologic association between cataract and hearing difficulties and provides new insights into the shared genetic architecture of these two disorders at the common variant level.

**Supplementary Information:**

The online version contains supplementary material available at 10.1186/s40246-024-00601-z.

## Introduction

In keeping with the aging of the population and the global burden of late-life disease, cataract and hearing difficulties have become the leading causes of blindness and hearing difficulties in the elderly [1, 2]. Although not life-threatening, they both have been shown to correlate with physical function, daily living, mental health, and dementia, leading to significant health burdens and economic costs worldwide [3–6]. Epidemiological studies of cataract and hearing difficulties comorbidity suggest that older adults appear at high risk of cataract and hearing difficulties [7], and hearing-impaired patients are 37% more likely to develop cataracts in Korean middle-aged and older adults [8]. However, no large-scale study has explored their epidemiologic relationship within other ethnic groups.

There is now substantial empirical evidence demonstrating that personal and environmental factors influence both cataracts and hearing difficulties. Several risk factors for cataracts are commonly present in hearing difficulties, including age, gender, ethnic origin, cigarette smoking, lower educational or socioeconomic status, diabetes, and hypertension [9, 10]. In addition, cataract may be linked to hearing difficulties due to similarities in embryonic development and pathological mechanisms. The lens and inner ear, as components of the sense organs of the vertebrate head, are both derived from the ectodermal placode and associated with the developing forebrain or hindbrain [11]. In addition, the opacity of the lens is a direct result of oxidative stress, while mounting evidence has also shown potential links between oxidative stress and cochlear pathology [1, 12]. Furthermore, alterations in visual and auditory sensory input correlate with the human cortex’s neuroplasticity, and intriguing biochemical and molecular pathways are needed to elucidate how cataract relates to hearing difficulties.

Cataracts and hearing difficulties are both expected to have significant heritability (h^2^). Twin and family studies support that genetic factors account for 35 to 58% in cataract susceptibility [13–15]. In recent years, genome-wide association studies (GWAS) have identified a large number of common genetic risk variants associated with cataract [15–17] and hearing difficulties [18–20] and indicated the heritability of hearing-related traits ranged from 7.6 to 13.7% [18]. However, to date, no study has investigated the genetic overlap between cataracts and hearing difficulties.

Unraveling the nature of these shared genetic risks is essential to identify underlying molecular mechanisms of complex human disorders, which might provide opportunities for preventive and therapeutic approaches to both conditions. A widely used method for assessing the genetic relationship between two disorders is to estimate genetic correlation (r_g_) by performing linkage disequilibrium (LD) score regressions [21]. However, the LD Score regression does not capture mixed-effect directions across shared genetic variants between complex phenotypes, limiting its application and interpretation [22]. The recently developed MiXeR [22] and conditional/conjunctional false discovery rate (cond/conjFDR) analysis [23] could quantify shared polygenic architecture between two phenotypes irrespective of the genetic correlation and detect single-nucleotide polymorphisms (SNPs) with mixed effect directions, hopefully improving the yield of existing GWASs. These approaches have enhanced the discovery of overlapping genetic variants between a wide range of complex human comorbidity, including psychiatric and neurological traits [24, 25].

The present study aimed to identify the epidemiologic association between cataracts and hearing difficulties and investigate whether cataracts share a genetic basis with hearing difficulties by utilizing the MiXeR and cond/conjFDR approach. By exploring the shared genetic architecture of cataracts and hearing difficulties, we aim to identify and characterize specific shared genomic loci and provide critical insights into their underlying pathophysiology.

## Materials and methods

### Participants for the epidemiologic analysis

UK Biobank is a prospective cohort study of approximately 500,000 participants aged 40–69 years recruited across the UK. Demographic information, visual examinations, medical history, and surgery history were ascertained through touch-screen questionnaires at the baseline recruitment visit.

UK Biobank has ethical approval from the North West Multi-centre Research Ethics Committee (11/NW/0382). This paper’s access to patient records is under UK Biobank Resource project #62,525. Written informed consent was obtained from all participants of this study. The study was conducted adhering to the tenets of the Declaration of Helsinki. We have also received the Ethics Review Exemption from Guangdong Provincial People’s Hospital, Guangdong Academy of Medical Sciences, for the research use of data and records that are all publicly available.

### Ascertainment of cataract and hearing difficulties

In the UK Biobank, cataract cases were defined through linkage to self-reported cataract operation (Field id: 20,004, code 1435) or/and a hospital record including a diagnosis code based on ICD-10 (ICD-10: H25 or H26), in accord with earlier GWAS research on cataracts [16]. Controls were participants who reported no eye disorders.

Hearing difficulties cases of the UK Biobank were identified by four hearing traits, which showed statistically significant heritability (h2) in earlier GWAS research on hearing difficulties [18]. The four traits used to determine self-reported hearing difficulties are (1) “Do you find it difficult to follow a conversation if there is background noise (such as TV, radio, children playing)?” (Field id: 2257, background noise problems); (2) “Do you have any difficulty with your hearing?” (Field id: 2247, hearing difficulty/problems); (3) “Do you use a hearing aid most of the time?” (Field id: 3393, hearing aid user); (4) “Do you get or have you had noises (such as ringing or buzzing) in your head or in one or both ears that last for more than five minutes at a time?” (Field id: 4803, tinnitus). Self-reporting hearing difficulties has previously been used in extensive cohort studies [26, 27]. Hearing difficulties cases were also defined through a hospital record including a diagnosis code based on ICD-10 (ICD-10: H90 or H91), that were classified as conductive and sensorineural hearing difficulties; ototoxic hearing difficulties; presbycusis; sudden idiopathic hearing difficulties, etc. We defined hearing difficulties as a positive response to any of the above questions and hospital record.

In sensitivity analysis, subjects responded “Yes” to both “background noise problems” and “hearing difficulty/problems” identified to detect hearing difficulties. Additionally, patients who had undergone cataract surgery were excluded, thus mitigating the impact of ocular surgery on the outcomes.

### Ascertainment of covariates

Demographic information included age, sex, and ethnicity (recorded as white and non-white). The ethnicity was self-reported and recorded as white and non-white (Asian, Black, Chinese, Mixed, or other ethnic groups), with the genetic ancestry of ‘White’ also confirmed by genotypes. Other covariates including educational qualifications, smoking, alcohol consumption, physical activity, and family history of severe depression were obtained through standardized questionnaires. Obesity was defined as BMI > 30 kg/m2. Diabetes mellitus, hypertension, and hyperlipidemia were defined by self-report, diagnoses, medications, or physical measurements. All demographic information is shown in Table S1.

### GWAS data sets

Summary statistics for investigating the genetic architecture of cataract was from the previous GWAS meta-analysis (67,844 cases and 517,399 controls), combining results from Genetic Epidemiology Research in Adult Health and Aging (GERA) and the UK Biobank cohorts [16]. The GERA project is a cohort of over 110,000 adult members participating in the Kaiser Permanente Medical Care Plan, Northern California Region, Research Program on genes, environment, and health.

Summary statistics for hearing difficulties were from the previous multi-trait analysis of hearing-related traits in the UK Biobank (*n* = 323,978), which supported 31 risk loci for hearing difficulty [18]. The multi-trait analysis was performed based on the above four hearing-related traits (background noise problems, hearing difficulty/problems, hearing aid user, and tinnitus), which had significant heritability. Individuals in all studies were predominantly of European ancestry, and detailed descriptions of sample recruitment and subsequent GWAS analyses are available in the original publications.

### Statistical analysis

#### Phenotypic analysis

Continuous variables were reported as mean (standard deviation) and compared through unpaired t-tests. Categorical variables were reported as numbers and percentages and compared through Pearson’s chi-square test. A matching process based on propensity score was done to equalize all potential prognostic factors mentioned above and to formulate a balanced 1:1 matched cohort study. Logistic regression models were used to estimate the adjusted OR and their 95% CI, adjusted for age and gender (model 1), or additionally adjusted for ethnicity, Townsend index, educational attainment, smoking, alcohol consumption, obesity, physical activity, and history of hypertension, diabetes, hyperlipidemia (model 2). All *P* values were two-sided, and a *P* value < 0.05 was considered significant. Analyses were performed using Stata version 13 (version 14.0; StataCorp).

#### Conditional quantile-quantile (QQ) plots

We constructed conditional QQ plots to visualize the putative overlap in SNPs between cataract and hearing difficulties after excluding SNPs within four regions with complex LD patterns (major histocompatibility complex regions: chr6:25119106–33,854,733; 8p23.1: chr8:7200000–12,500,000; the MAPT region: chr17:40000000–47,000,000; and the apolipoprotein E region: chr19:44909039–45,912,650) [28]. Enrichment exists when the proportion of SNPs associated with a primary phenotype (e.g., cataract) increases as a function of the strength of the association with a secondary phenotype (e.g., hearing difficulties) [24]. Each QQ plot reveals the distribution of *P* values for the primary phenotype conditioning on the significance of association with the secondary phenotype at *P* < 0.10, *P* < 0.01, *P* < 0.001, and *P* < 0.0001.

#### MiXeR

Polygenic overlap between cataract and hearing difficulties, irrespective of genetic correlation between selected phenotypes, was evaluated by MiXeR [22]. Based on the Akaike information criterion, MiXeR evaluated model fitting based on the power of existing summary statistics. First, we constructed a univariate mixture model to estimate the number of disorder-influencing variants. Next, we performed a bivariate model additive genetic associations with two traits as a mixture of 4 bivariate Gaussian components, (i) SNPs not influencing either phenotype; (ii & iii) SNPs uniquely influencing either the primary or secondary phenotype; and (iv) SNPs influencing both phenotypes. Last, we used MiXeR to calculate a Dice coefficient, a ratio of shared variants to the total number of variants, to evaluate the polygenic overlap. Results were presented as Venn diagrams displaying the proportion of unique and shared SNPs.

#### Conditional and conjunction false discovery rate

The conditional/conjunctional false discovery rate (cond/conjFDR) approach was applied to increase genetic discovery power and identify specific shared loci between cataract and hearing difficulties [23]. Like standard GWAS analysis, the condFDR/conjFDR method does not operate on a causal level but identifies LD proxies of the underlying causal variants. In pleiotropy analysis, FDR reflects the possibility of non-pleiotropy for an SNP. The condFDR approach builds on Bayesian statistics and increases the power to identify loci associated with a primary phenotype (e.g., cataract) by leveraging associations with a secondary phenotype (e.g., hearing difficulties) [29]. Thus, this method re-ranks test statistics using the associations between variants and the secondary phenotype and re-calculates the associations between these variants and the primary phenotype. Inverting the roles of primary and secondary phenotypes yields the inverse condFDR value. ConjFDR is an extension of condFDR and can detect loci jointly associated with two phenotypes [30]. After repeating condFDR for both traits, we applied conjFDR analysis to identify shared genetic loci between cataract and hearing difficulties. ConjFDR is defined as the maximum of the two condFDR values, providing a conservative estimate of the false discovery rate for an SNP association with both phenotypes. We examined the significance and directionality of allelic association for identified loci in independent cohorts using lead SNPs. Overall, FDR thresholds of 0.01 and 0.05 were chosen for conditional and conjunctional FDR, respectively, consistent with previous publications [24, 31].

### Genomic loci definition and functional annotation

The results of each analysis were filtered as follows [31]. First, we filtered the lists of significant SNPs by their LD structure (r2-value) as observed in the 1000 Genomes dataset and report only the most significant result per annotated gene. We considered an SNP an independent finding if LD r2 < 0.2 with all other SNPs. Second, we further filtered the list of significant SNPs for novelty. A locus that was not physically overlapping with findings from the original GWASs or National Human Genome Research Institute–European Bioinformatics Institute GWAS Catalog (https://www.ebi.ac.uk/gwas/home) was considered novel. Candidate SNPs were functionally annotated to characterize their biological significance and highlight putative causal genes. Genes were mapped using three strategies: (i) positional mapping: to genes within 10 kb distance; (ii) expression quantitative trait locus (eQTL) mapping; and (iii) chromatin interaction mapping: to genes with which they are predicted to interact by 3D modeling of chromatin structure physically [32].

To gain insights into the biological mechanisms, we checked the putative genes for any known association with cataract or hearing difficulties within public expression datasets obtained from the NCBI GEO (https://www.ncbi.nlm.nih.gov/geo/). From NCBI GEO, we interrogated the microarray and RNA-seq datasets of human eye and human lens cells (GSE3023 and GSE2256) and microarray data from cochlear inner and outer hair cells from mice (GSE56866). Previous GWAS studies have well illustrated considerations for using mouse cochlea rather than human for hearing difficulties [18] and discussed in the limitation. Differential expression for each dataset was interrogated using the GEO2R software using a moderated t statistic.

## Results

### Phenotypic association between cataract and hearing difficulties

A total of 142,069 participants at the baseline were included in the present study, with a mean age of 56.71 ± 8.12 years and 54.90% females. Of these, 10,436 (7.35%) were diagnosed with cataracts, and 61,352 (55.15%) were reported with hearing difficulties. Table 1 summarizes the baseline characteristics of the study participants stratified by cataracts and hearing difficulties.

**Table 1:** Descriptive characteristics for the overall population recruited from the UK Biobank, stratified by cataract and hearing difficulties at baseline

Baseline characteristics	Total	Sample with cataract	Controls for cataract	Sample with hearing difficulties	Controls for hearing difficulties
**Number**	142,069	10,436	131,633	61,352	49,887
**Age, years**	56.71 (8.12)	62.38 (6.01)	56.26 (8.09)	57.99 (7.79)	55.45 (8.23)
**Gender, %**					
Female	77,996 (54.90)	5,447 (52.20)	72,549 (55.11)	29,844 (48.64)	29,632 (59.40)
Male	64,072 (45.10)	4,988 (47.80)	59,084 (44.89)	31,507 (51.36)	20,255 (40.60)
**Ethnicity, %**					
White	129,338 (91.04)	9,375 (89.83)	119,963 (91.13)	56,794 (92.57)	44,617 (89.44)
Non-white	12,731 (8.96)	1,061 (10.17)	11,670 (8.87)	4,558 (7.43)	5,270 (10.56)
**Townsend index**	-1.01 (3.06)	-0.93 (3.27)	-1.02 (3.05)	-1.01 (3.08)	-1.07 (2.94)
**Education, %**					
College/University degree	48,187 (33.92)	2,828 (27.10)	45,359 (34.46)	20,011 (32.62)	18,690 (37.46)
Others	93,882 (66.08)	7,608 (72.90)	86,274 (65.54)	41,341 (67.38)	31,197 (62.54)
**Smoking status, %**					
Never	77,874 (55.02)	5,240 (50.66)	72,634 (55.37)	31,453 (51.47)	29,267 (58.83)
Prior/current	63,652 (44.98)	5,103 (49.34)	58,549 (44.63)	29,655 (48.53)	20,485 (41.17)
**Drinking status, %**					
Never	7,109 (5.01)	722 (6.97)	6,387 (4.86)	2,821 (4.60)	2,623 (5.26)
Prior/current	134,754 (94.99)	9,643 (93.03)	125,111 (95.14)	58,461 (95.40)	47,223 (94.74)
**Obesity, %**					
No	106,333 (75.43)	7,371 (71.25)	98,962 (75.77)	45,144 (74.17)	38,307 (77.39)
Yes	34,627 (24.57)	2,974 (28.75)	31,653 (24.23)	15,725 (25.83)	11,193 (22.61)
**History of hypertension, %**					
No	37,704 (26.54)	1,946 (18.65)	35,758 (27.16)	15,304 (24.94)	13,992 (28.05)
Yes	104,365 (73.46)	8,490 (81.35)	95,875 (72.84)	46,048 (75.06)	35,895 (71.95)
**History of diabetes, %**					
No	134,005 (94.32)	8,905 (85.33)	125,100 (95.04)	57,444 (93.63)	47,493 (95.20)
Yes	8,064 (5.68)	1,531(14.67)	6,533 (4.96)	3,908 (6.37)	2,394 (4.80)
**History of hyperlipidemia, %**					
No	77,022 (54.21)	4,517 (43.28)	72,505 (55.08)	31,808 (51.85)	28,374 (56.88)
Yes	65,047 (45.79)	5,919 (56.72)	59,128 (44.92)	29,544 (48.15)	21,513 (43.12)
**Physical activity, %**					
Not meeting recommendation	20,753 (18.04)	1,555 (19.42)	19,198 (17.94)	9,454 (19.03)	7,021 (16.91)
Meeting recommendation	94,279 (81.96)	6,452 (80.58)	87,827 (82.06)	40,235 (80.97)	34,500 (83.09)

Values are means (standard deviation) or percentages and are standardized to the age distribution of the study population

Values of polytomous variables may not sum to 100% due to rounding

Before matching, the adjusted OR of hearing difficulties was 2.12 (95% confidence interval [CI] 2.00-2.25, *P* < 0.001) among participants with cataracts at the baseline after controlling age and gender, compared with those with no eye disorder. The association remained robust when additionally controlling ethnicity, Townsend index, educational attainment, smoking, alcohol consumption, obesity, history of hypertension, diabetes, hyperlipidemia, and physical activity (OR 2.12, 95% CI 1.98–2.27, *P* < 0.001).

After matching 1:1 on the propensity score, we created 4,484 pairs (*n* = 8,968) with a documented cataract to those without eye disorder. All measured baseline differences in the unmatched sample (age, gender, ethnicity, Townsend index, educational attainment, smoking, alcohol consumption, obesity, history of hypertension, diabetes, hyperlipidemia, and physical activity) were adequately balanced after matching. Tests for multicollinearity between the above covariates indicated that all correlations were below 0.5. The elevated risk of hearing difficulties was still statistically significant for participants with cataracts compared with those with no eye disorder (OR 2.03, 95% CI 1.86–2.23, *P* < 0.001, Table 2). The relation between cataracts and hearing difficulties did not vary by age (p_interaction_ = 0.23) or gender (p_interaction_ = 0.83).

**Table 2:** Cataract and risk of hearing difficulties in the UK Biobank Study

Cataract status	Cases	Model 1		Model 2	
		OR (95% CI)	*P* value	OR (95% CI)	*P* value
Unmatched analysis					
**Controls**	131,633	1.00 (ref)	-	1.00 (ref)	-
**Cataract**	10,436	**2.12 (2.00-2.25)**	**< 0.001**	**2.12 (1.98–2.27)**	**< 0.001**
**Matched analysis**					
**Controls**	4,484	1.00 (ref)	-	1.00 (ref)	-
**Cataract**	4,484	**2.04 (1.86–2.23)**	**< 0.001**	**2.03 (1.86–2.23)**	**< 0.001**
**Sensitivity analysis** ^**1**^					
**Controls**	4,484	1.00 (ref)	-	1.00 (ref)	**-**
**Cataract**	4,484	**1.95 (1.76–2.17)**	**< 0.001**	**1.95 (1.75–2.17)**	**< 0.001**
**Sensitivity analysis** ^**2**^					
**Controls**	2,072	1.00 (ref)	-	1.00 (ref)	**-**
**Cataract**	2,072	**1.98 (1.69–2.31)**	**< 0.001**	**1.99 (1.71–2.33)**	**< 0.001**

OR = odds ratio; CI = confidence interval. Bold values denote statistical significance at *P* < 0.05 level. Model 1 has been adjusted for age and gender. Model 2 has been adjusted for age, gender, ethnicity, Townsend index, educational attainment, smoking, alcohol consumption, obesity, physical activity, and history of hypertension, diabetes, and hyperlipidemia

Sensitivity analysis^1^ was limited to subjects with hearing difficulties who responded affirmatively to both “background noise problems” and “hearing difficulty/problems”

Sensitivity analysis^2^ was confined to subjects with no history of cataract surgery

In sensitivity analyses that used subjects who responded “Yes” to both “background noise problems” and “hearing difficulty/problems” to detect hearing difficulties (*n* = 3,220, 52.79%), the results did not significantly differ compared with the primary analyses(OR 1.95, 95% CI 1.75–2.17, *P* < 0.001, Table 2). Similar results for the association between cataracts and hearing difficulties were seen after excluding individuals who had undergone cataract surgery (OR 1.99, 95% CI 1.71–2.33, *P* < 0.001, Table 2).

### Genetic overlap between cataracts and hearing difficulties

**Fig. 1:**
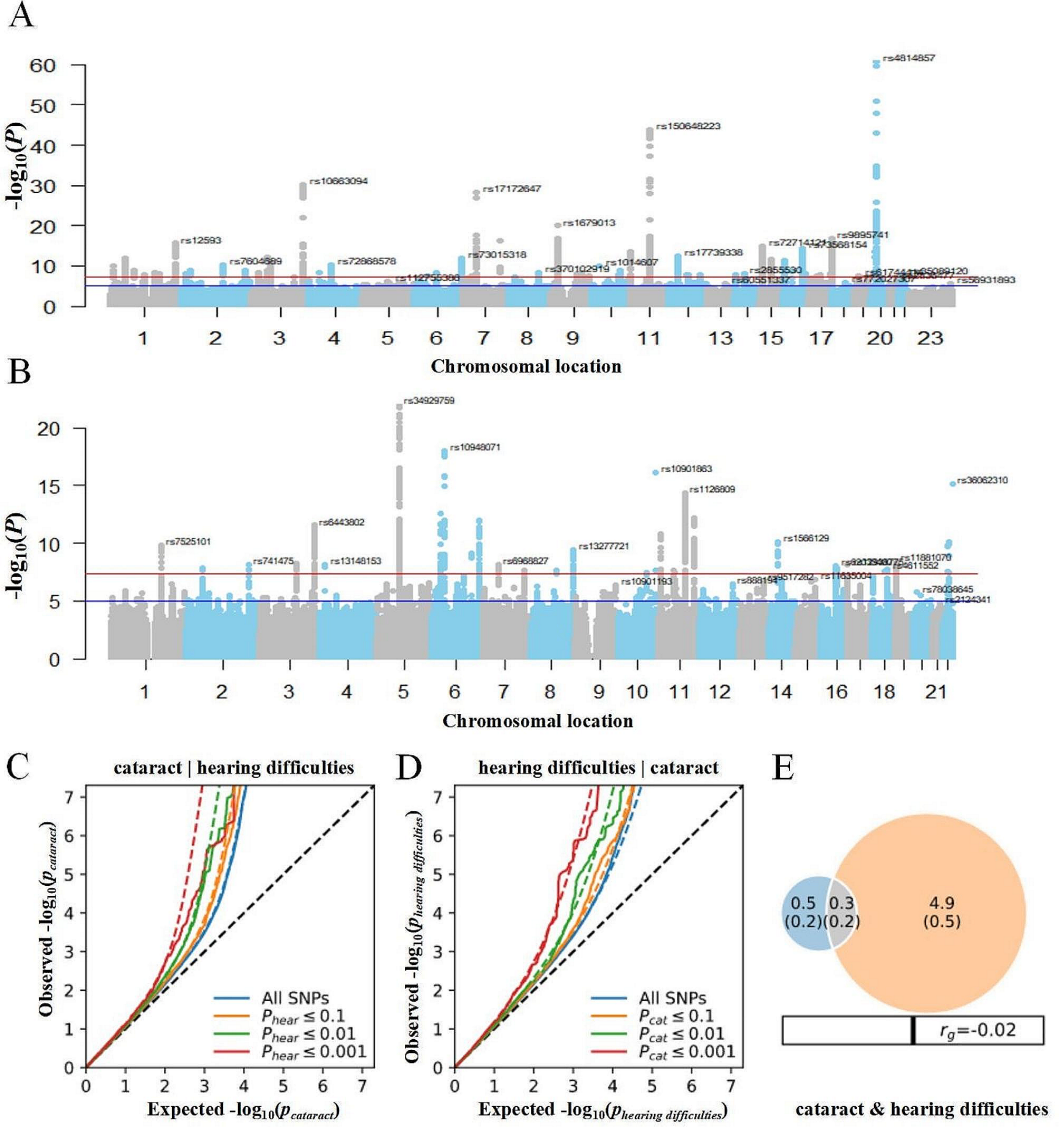
Manhattan plots and conditional quantile-quantile (QQ) plots showed genetic vulnerabilities of cataract and hearing difficulties. (**A**) Manhattan plots displaying previous GWAS Results for cataract (GERA + UK Biobank) with the *p* values of all SNPs [16]. (**B**) Manhattan plots showing previous GWAS Results for hearing difficulties phenotypes (UK Biobank) [18]. The threshold for genome-wide significance (*p* < 5 × 10^− 8^) is indicated by a red dotted line. Loci that reached genome-wide significance in phenotypes are annotated with gene symbols. (**C**, **D**) QQ plots are shown of observed versus expected -log10 *P* values in the primary phenotype (e.g.cataracts) as a function of significance of association with a secondary phenotype (e.g., hearing difficulties). (**E**) The Venn diagram depicts the estimated number of trait-influencing variants shared (gray) between cataracts (left circle) and hearing difficulties phenotypes (right circle). The number of trait-influencing variants in thousands is shown, with the standard error in thousands provided. The size of the circles reflects the polygenicity of cataract or hearing difficulties, with larger circles corresponding to greater polygenicity and vice versa

Given the observation in the epidemiological analysis, we subsequently focused on confirming and identifying polygenic overlap between cataracts and hearing difficulties. The corresponding Manhattan plots of these two phenotypes are presented in Fig. 1A and B. The stratified conditional QQ plots showed SNPs enrichment for cataract as a function of the significance of associations with hearing difficulties and vice versa, indicating the existence of polygenic overlap (Fig. 1C,D).

Bivariate MiXeR analysis revealed a moderate polygenic overlap of cataracts influencing variants with hearing difficulties (Fig. 1E). 37.5% of variants associated with cataracts (300 of 800; Dice coefficient = 0.08, SD = 0.06) may contribute to the risk of hearing difficulties. MiXeR also revealed a higher polygenicity in hearing difficulties than in cataracts, with 4900 variants associated with hearing difficulties but not cataracts. The log-likelihood plot, illustrating the relationship between the GWAS test statistics, is presented in the Supplemental data (Figure S1).

The cond/conjFDR analysis identified 7066 genomic loci jointly associated with cataract and hearing difficulties (Figure S2, Table S2), of which 6 significant genomic loci (LD r2 < 0.2 with all other SNPs) showed *P* value < 1 × 10^− 4^ for both phenotypes (Table 3). Of the 6 significant genomic loci, 3 and 5 novel loci were discovered for cataract or hearing difficulties, respectively. The shared loci showed mixed directions of allelic associations, with 3 of 6 loci (50%) showing concordant associations with cataract and hearing difficulties. To investigate the consistency of genetic effects in an independent sample, we utilized a second GERA data sets for hearing difficulties to replicate [33]. Of 6 shared genomic loci identified between cataract and hearing difficulties, 2 loci (rs12295166, rs9912530) were also replicated with a consistent direction of effect at cond/conjFDR < 0.05.

**Table 3:** Independent loci reaching statistical significance false discovery rate shared between cataracts and hearing difficulties in the hg19 human genome reference

SNP	Chr	Pos	Nearest gene	Alleles A1/A2	MAF	Cond/conjFDR
rs9290737	3	181,979,272	LINC01206	A/G	0.32	6.08E-05
rs4709714	6	163,801,031	QKI	C/T	0.56	6.97E-05
rs55728135	7	43,692,493	STK17A	A/G	0.21	7.76E-05
rs12295166	11	88,976,157	TYR	T/C	0.37	7.81E-05
rs9912530	17	44,836,302	NSF	T/C	0.71	9.82E-05
rs613872	18	53,210,302	TCF4	G/T	0.83	0.000187727

Note: Gene context for each significant independent SNP was examined in the NCBI database (http://www.ncbi.nlm.nih.gov/). Abbreviations: SNP, single nucleotide polymorphism; MAF, minor allele frequency; Cond/conjFDR, conditional/conjunctional false discovery rate

### Biological insights from shared loci by cataracts and hearing difficulties

The 6 shared loci were mapped to 6 genes (*LINC01206*, *QKI, STK17A*, *TYR*, *NSF*, and *TCF4*) to explore their biological relationships. The role of *Qki* has been well demonstrated for the transcriptional activation of genes involved in cholesterol biosynthesis of the eye lens in a tissue-specific manner [34]. Besides, analysis of QKI-deficient mice also revealed that QKI expression in cochlear glial cells is essential for the myelination of spiral ganglion neurons and auditory nerve fibers, as well as for normal hearing [35]. The *TYR* gene is a member of the TYRosinase-related protein families, and the TYRosinase is the rate-limiting enzyme in the melanin synthesis [36]. Mutations in this gene have been correlated with hearing difficulties in humans and mice [37, 38], as melanin is vital for preserving the cochlea against aging. *TYR* mutants may also result in oculocutaneous albinism, pigment dispersion syndrome, or pigmentary glaucoma in the eye [39, 40]. The *NSF* gene encodes a molecule essential for intracellular vesicle transport and membrane fusion and plays a role in synaptic transmission. The expression of *Nsf* has been detected in both lateral line hair cells and afferent neurons of the zebrafish lateral line organ, indicating a role in maintaining synaptic contacts between hair cells and afferent neurons [41]. In addition, TCF4 showed a significant association with endothelial corneal dystrophy and abnormal retina morphology in vitro system and early adult mice [42].

**Fig. 2:**
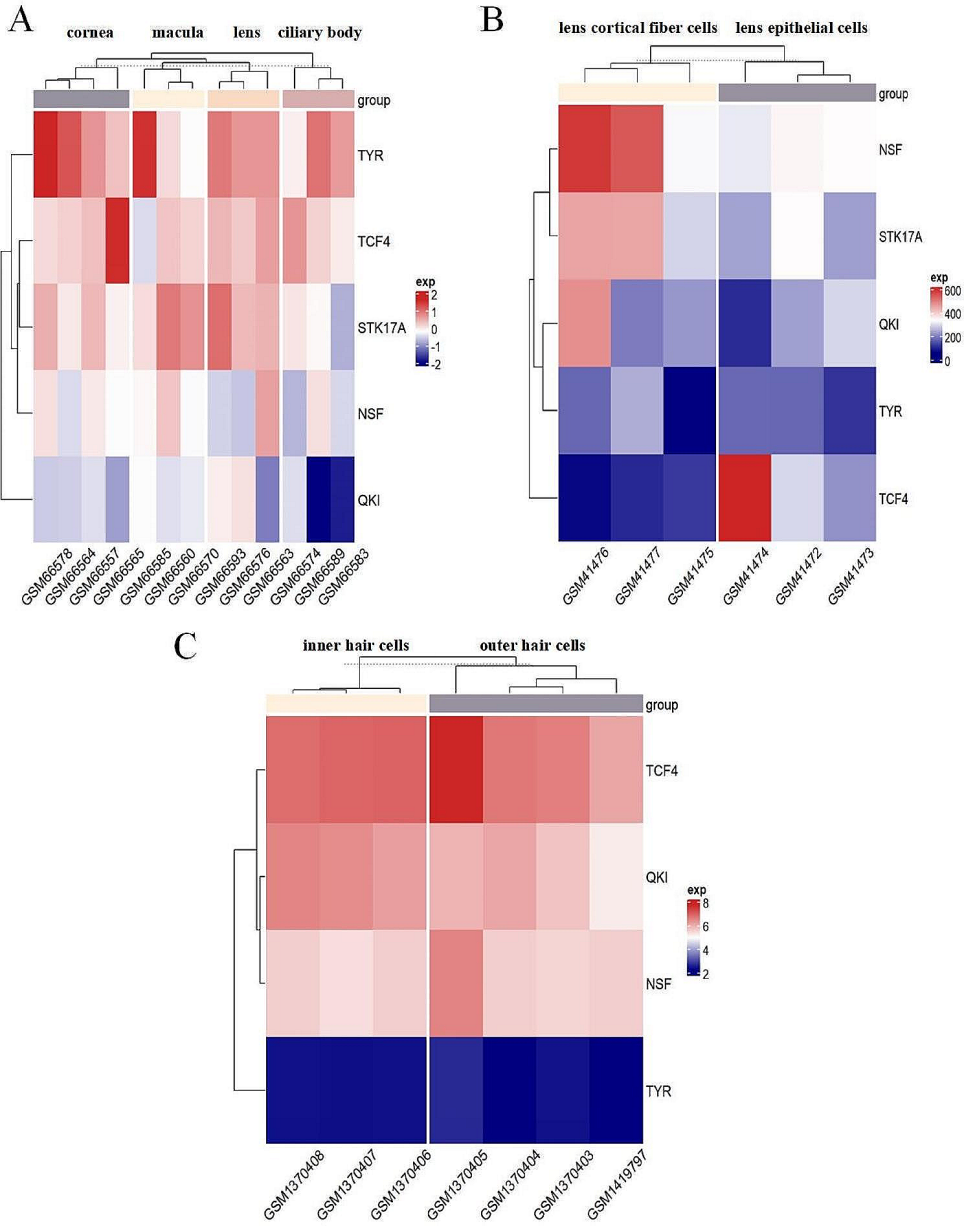
The GEO dataset verifies the expression profiles in lens and cochlear hair cells for genes mapped to lead SNPs. (**A**) The heat map for human whole eye globes from autopsy donors (age range = 30–85 y) (GSE3023). (**B**) The heat map for human lens cells compares lens epithelial cells with lens cortical fiber cells (age over 50 y) (GSE2256). (**C**) The heat map for cochlear inner and outer hair cells from mice (25–30 d old) (GSE56866)

We performed tissue enrichment analysis for the 5 protein-coding genes using transcriptome profiling of lens [43] and cochlear hair cells [44]. Analysis of these datasets confirmed selective expression in the lens for 4 of the 5 genes above (*STK17A*, *TYR*, *NSF*, and *TCF4*), with *TCF4* highly expressed in lens epithelial cells, while *STK17A* and *NSF* mainly expressed in lens cortical fiber cells (Fig. 2A,B). This analysis also revealed low but particular expression in hair cells for QKI, NSF, and TCF4 (Fig. 2C). Thus, we confirmed that *NSF* and *TCF4* are jointly associated with cataract and hearing difficulties and expressed selectively in lens and cochlear hair cells.

## Discussion

This study assessed the phenotypic association and identified a shared genetic basis between cataracts and hearing difficulties. Specifically, we (1) confirmed that cataracts among middle-aged and older adults were independently associated with hearing difficulties in the UK population; (2) revealed polygenic overlap and 6 shared genomic loci between cataracts and hearing difficulties; and (3) suggested QKI, STK17A, TYR, NSF, and TCF4 genes likely contribute to the pathophysiology of cataracts and hearing difficulties. These findings offer insights into the genetic basis of the comorbidity between cataracts and hearing difficulties, and highlight several putative genes for experimental validation with potential for drug discovery and personalized treatment.

To the best of our knowledge, this is the first in-depth dissection of the link between cataracts and hearing difficulties, from phenotypic to genetic associations. Only one study reported various opacities of the lens were more common in the hearing-impaired group than in the normal group [8], suggesting patients with hearing impairment should be checked for the presence of cataract. Klein et al. reported risk factors, including smoking and heavy drinking, were associated with concurrent age-related cataract and hearing difficulties [7]. Our results confirmed the cross-sectional association between cataracts and hearing difficulties, which is not entirely explained by proxies of major risk factors. Although unmeasured confounders cannot be ruled out, one possible explanation is the common genetic factors underlying both phenotypes. We further illustrated the genetic association of both phenotypes with genome-wide loci shared between cataracts and hearing difficulties were identified.

Our findings demonstrate a small genetic overlap of cataract-influencing variants with hearing difficulties, with mixed directions of effect and minimal genetic correlation. In terms of SNPs distribution, rs9290737, rs4709714, and rs9912530 are intergenic SNPs, while the remaining three rs55728135, rs12295166, and rs613872 are intronic. The intronic SNP rs55728135, a locus adjacent to the STK17A gene, is expressed in several tissues, including coronary artery, left ventricle, atrial appendage, etc. [45]. Additionally, targeting STK17A gene may attenuate ROS and protect against myocardial ischemia-reperfusion injury [46]. A meta-analysis has reported that histone marks associated with enhancers, namely H3K4me1 and H3K27ac, have been detected in primary foreskin melanocyte cells, coinciding with two variants exhibiting high linkage disequilibrium (LD) with rs1042602 [47]. Mutations in the TYR gene may cause oculocutaneous albinism [48]. Several studies have shown that the TCF4 gene variant rs613872 is associated with increased affectations and clinical disease phenotypes [49], and also correlates with Fuchs’ endothelial corneal dystroph [50–52]. It is unsurprising given their clinical differences reflecting distinct pathological mechanisms. The reason for the comorbidity of cataracts and hearing difficulties is complex and not explained solely by a single pathway. However, a proportion of this overlap is likely driven by *Qki*, which is involved in cholesterol biosynthesis of the eye lens [34], glia dysfunction and demyelination in hearing difficulties [35]. Mitochondrial dysfunction and oxidative stress potentially result from *TCF4* mutations and are correlated with aging, which may also explain the observed association [53]. We note that *TCF4* was confirmed to have high lens-enriched expressions and in hair cells in our study.

Some limitations of the study should be mentioned. First, our analysis is based on self-reported hearing difficulty, likely less accurate than a quantitative hearing assessment. Nevertheless, the GWAS analysis of objective measures of hearing using “Digits in Noise” protocol was not sufficient to yield heritability in a previous report [54]. Second, we could not confirm the reason and the age of onset of cataract or hearing difficulty in the UK Biobank cohort, making an accurate classification of hearing difficulties a challenge. Third, there is currently a lack of adequately powered studies to replicate our results, and several loci have not yet been replicated. It is, therefore, unsurprising that only 2 loci, rs12295166, and rs9912530, were replicated. Finally, the putative genes remain data-driven hypotheses that need to be functionally validated in the future. As greater attention is focused on the aging population’s health, future research is necessary to elucidate the mechanistic pathways.

Previous GWAS studies have well illustrated considerations for using mouse cochlea rather than humans for hearing difficulties [18]. Firstly, novel deafness-related genes and regulators of inner ear development have been successfully identified using mice [55–57]. Secondly, non-coding gene regulatory elements are evolutionarily conserved between humans and mice [58]. Thirdly, human cochleae are not readily available for biopsies and are rarely surgically removed.

In summary, our results demonstrate that cataracts and hearing difficulties are correlated and reveal shared polygenicity. The genetic overlap between cataract and hearing difficulties may partly explain the phenotypic association found in the epidemiological analysis. The identified shared locus potentially lead to the aging biology involving the neurodevelopment process of brains for both phenotypes.

## Electronic supplementary material

Below is the link to the electronic supplementary material.

Supplementary Material 1

Supplementary Material 2

## Data Availability

UK Biobank provided all phenotypic data analyzed herein. A guide to access is available from the UK Biobank website (http://www.ukbiobank.ac.uk/register-apply/). The combined (GERA & UKB) meta-analysis GWAS summary statistics for cataracts are available from the NHGRI-EBI GWAS Catalog (https://www.ebi.ac.uk/gwas/downloads/summary-statistics), study accession number GCST90014268. The GWAS summary statistics for hearing difficulties (UKB) are available from the NHGRI-EBI GWAS Catalog study accession number GCST90012115. The second GWAS summary statistics for hearing difficulties (GERA) are available from the NHGRI-EBI GWAS Catalog study accession number GCST003763. Additionally, public expression datasets were obtained from the NCBI GEO (https://www.ncbi.nlm.nih.gov/geo/). From NCBI GEO, we interrogated the microarray and RNA-seq datasets of human eye and human lens cells (GSE3023 and GSE2256) and microarray data from cochlear inner and outer hair cells from mice (GSE56866). We used publicly available software for the analyses. The software programs used are listed and described in the Methods. MiXER: GitHub. precimed/mixer. https://github.com/precimed/mixer. cFDR: GitHub. KehaoWu/GWAScFDR. https://github.com/KehaoWu/GWAScFDR.

## Abbreviations

CI: confidence interval
Cond/conjFDR: conditional/conjunctional false discovery rate
GERA: Genetic Epidemiology Research in Adult Health and Aging
GO: gene ontology
GWAS: genome-wide association studies
OR: odds ratio
LD: linkage disequilibrium
MAF: minor allele frequency
Q-Q: quantile–quantile
SNPs: single nucleotide polymorphisms
