# Correction: Epidemiologic association and shared genetic architecture between cataract and hearing difficulties among middle-aged and older adults

**DOI:** 10.1186/s40246-025-00737-6

**Published:** 2025-03-13

**Authors:** Xiayin Zhang, Shan Wang, Shunming Liu, Zijing Du, Guanrong Wu, Yingying Liang, Yu Huang, Xianwen Shang, Yijun Hu, Zhuoting Zhu, Wei Sun, Xueli Zhang, Honghua Yu

**Affiliations:** 1 Department of Ophthalmology, Guangdong Eye Institute, Guangdong Provincial People’s Hospital, Guangdong Academy of Medical Sciences, Southern Medical University, Guangzhou, China; 2 Guangdong Cardiovascular Institute, Guangdong Provincial People’s Hospital, Guangdong Academy of Medical Sciences, Guangzhou, China; 3 Centre for Eye Research Australia, Royal Victorian Eye and Ear Hospital, VIC East Melbourne, Australia; 4 Department of Ophthalmology, Guangzhou First people’s Hospital, Guangzhou, China; 5 Guangdong Provincial Key Laboratory of Artificial Intelligence in Medical Image Analysis and Application, Guangzhou, China

**Correction: Human Genomics (2024) 18:39**

10.1186/s40246-024-00601-z

The authors wish to note a correction to a Funding source number presented in the original article.

‘20220610092’ should instead read as ‘202206010092’.

