# Supplementary material for: Epidemiologic association and shared genetic architecture between cataract and hearing difficulties among middle-aged and older adults
Source: Hum Genomics. 2024 Apr 17;18:39. doi: 10.1186/s40246-024-00601-z (PMC11022469; doi:10.1186/s40246-024-00601-z)
Supplement: Supplementary file 1 — Supplementary Material 1 [file 40246_2024_601_MOESM1_ESM.doc]

**Supplementary Online Content**

# Epidemiologic association and shared genetic architecture between cataract and hearing difficulties among middle-aged and older adults of European ancestry

**Table S1.** UK biobank showcase variables used in the phenotypic analysis.

**Figure S1**. Log-likelihood curves plotting the negative log-likelihood function (lower values correspond to better model fit) against the π12 parameter (number of influencing variants shared between cataract and hearing difficulties).

**Figure S2**. Common genetic variants jointly associated with cataract and hearing difficulties at cond/conjFDR < 0.01.

**Table S1. UK biobank showcase variables used in the phenotypic analysis.**

| **Measure** | **Field ID** | **Time** | **Description** |
| --- | --- | --- | --- |
| **Cataract cases and controls** | | | |
| Cataract operation | 20004(code 1435) | Baseline assessment | Ever underwent cataract operations. If the participant was uncertain of the type of operation they had undergone, then they described it to the interviewer (a trained nurse) who attempted to place it within the coding tree. |
| Hospital records for cataracts | 41270 (code H25 /H26) | Baseline assessment | Hospital in-patient records with cataract as main or any secondary diagnoses based on the 10th edition of the WHO International Classification of Diseases (ICD-10). |
| **H****earing difficulties cases and controls** | | | |
| Background noise problems | 2257 | Baseline assessment | “Do you find it difficult to follow a conversation if there is background noise (such as TV, radio, children playing)?” |
| Hearing difficulty/problems | 2247 |  | “Do you have any difficulty with your hearing?” (Field id: 7, ) |
| Hearing aid user | 3393 |  | “Do you use a hearing aid most of the time?” |
| Tinnitus | 4803 |  | “Do you get or have you had noises (such as ringing or buzzing) in your head or in one or both ears that lasts for more than five minutes at a time?” |
| Hospital records for hearing difficulties | 41270 (code H90 /H91) | Baseline assessment | Hospital in-patient records with hearing difficulties as main or any secondary diagnoses based on the 10th edition of the WHO International Classification of Diseases (ICD-10). |
| **Demographic information** | | | |
| Age | 21003 | Baseline assessment | Refer to the age of the participant on the day they attended an Assessment Centre, year. |
| Sex | 31 | Baseline assessment | Sex of participant |
| Ethnic background | 21000 | Baseline assessment | Self-reported and recorded as white and non-white (Asian, Black, Chinese, Mixed, or other ethnic groups). |
| Townsend deprivation index | 189 | Baseline assessment | Townsend deprivation index calculated immediately prior to participant joining UK Biobank based on the preceding national census output areas. Each participant is assigned a score corresponding to the output area in which their postcode is located. |
| Physical activity levels | 22036 | Baseline assessment | Indicates whether a person met the 2017 UK Physical activity guidelines of 150 minutes of walking or moderate activity per week or 75 minutes of vigorous activity. |
| Smoking status | 20116 | Baseline assessment | This field summarises the current/past smoking status of the participant. |
| Alcohol consumption | 20117 | Baseline assessment | Alcohol drinker status recorded as current/previous or never. |
| Education attainment | 6138 | Baseline assessment | Touchscreen question "Which of the following qualifications do you have? (You can select more than one)". |
| Obesity | 21001 | Baseline assessment | Body mass index (BMI) value is constructed from height and weight. Obesity was defined as BMI > 30 kg/m^2^. |
| Hypertension | 20002 (code 1065, 1072) | Baseline assessment | Self-reported hypertension. |
|  | 6153  (code 2) |  | Use of antihypertensive drugs. |
|  | 4080 |  | Average systolic blood pressure of at least 130mmHg. |
|  | 4079 |  | Average diastolic blood pressure of at least 80mmHg. |
| Diabetes mellitus | 2443 | Baseline assessment | Doctor-diagnosed diabetes mellitus. Touchscreen question "Has a doctor ever told you that you have diabetes?" |
|  | 20003 |  | The use of anti-hyperglycemic medications. |
|  | 6153  (code 3) |  | The use of insulin. |
|  | 30750 |  | Glycated hemoglobin level measured by HPLC analysis on a Bio-Rad VARIANT II Turbo (≥ 48 mmol/mol). |
| Hyperlipidemia | 20002  (code 1473) | Baseline assessment | Self-reported hyperlipidemia. |
|  | 6153 |  | The use of statins. |
|  | 20003 |  | The use of hyperlipidemia-related medication. |
|  | 30690 |  | Blood cholesterol level Measured by CHO-POD analysis on a Beckman Coulter AU5800(≥ 6.21 mmol/L). |


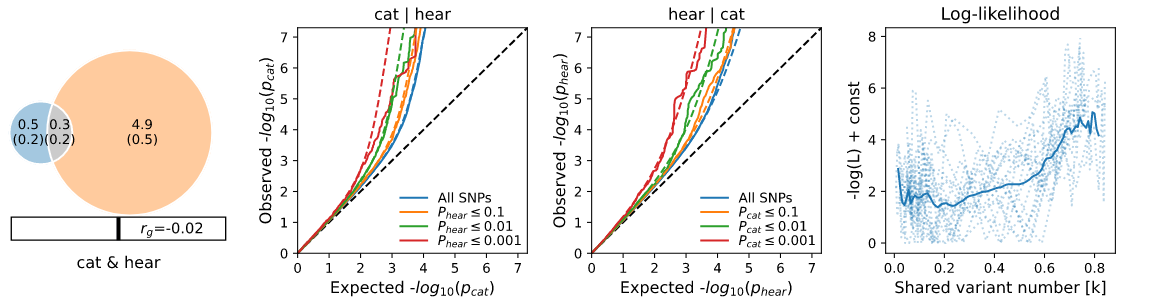


**Figure S1. Log-likelihood curves plotting the negative log-likelihood function (lower values correspond to better model fit) against the π_12_ parameter (number of influencing variants shared between cataract and hearing difficulties).**


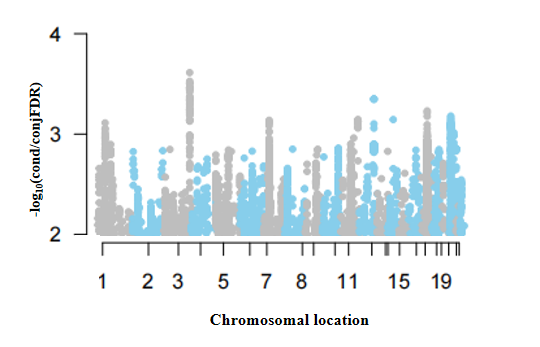


**Figure S2. Common genetic variants jointly associated with cataract and hearing difficulties at cond/conjFDR < 0.01.** Manhattan plots show the -log10 transformed cond/conjFDR values for each SNP on the y-axis and chromosomal positions along the x-axis. The significant shared signals are provided in Table 3. Cond/conjFDR, conditional/conjunctional false discovery rate.
